# Prolonged noise exposure-induced auditory threshold shifts in wistar rats: Proposal of an experimental exposure protocol based on noise dose

**DOI:** 10.1016/j.bjorl.2025.101570

**Published:** 2025-04-02

**Authors:** Ana Carolina Odorizzi Zica, Gabriela Ribeiro Novanta, Maria Luiza Queiroz Sampaio, Rafael Rocha de Andrade, Lucieny Silva Martins Serra, André Luiz Lopes Sampaio

**Affiliations:** a Universidade de Brasília (UnB), Brasília, DF, Brazil; b Universidade Católica de Brasília, Brasília, DF, Brazil; c University of Brasília (UnB), Faculdade de Medicina, Laboratório de Ensino e Pesquisa em Otorrinolaringologia, Brasília, DF, Brazil

**Keywords:** Hearing loss, Otoacoustic emissions, Noise and wistar rats

## Abstract

**Objective:**

The aim of the present study was to evaluate the effects of different doses of broadband white noise in a group of Wistar rats.

**Methods:**

The sample consisted of 6 male Wistar rats. The animals went through sequential exposures at the intensity of 95 dB for 60 min, 100 dB for 60 min and 100 dB for 120 min. Audiological evaluation was performed by Distortion Product Otoacoustic Emissions (DPOAE) exams at frequencies from 2 to 12 KHz, after each exposure/pause protocol. Statistical analysis was performed based on the values found in the DPOAE and Signal-to-Noise Ratio (S/N) in the nine exams performed over 50 days of research.

**Results:**

The study concluded that there was a compound threshold shift induced by long-term sequential noise exposure at the intensity of 100 dB for 60 min and for 120 min, with a “plateau/’ observed in the DPOAE and S/N results that coincide with the exposure period of 6 days at the intensity of 100 dB for 120 min.

**Conclusion:**

Thus, we established the proposal of an exposure protocol for research with wistar rats exposed to noise.

## Highlights

• Occupational noise being the main cause of sensorineural hearing loss in adults.
• Short-term noise exposures that possibly generated temporary damage.
• Intensity and time are determining factors in exposure to noise.

## Levels of evidence

The Oxford Centre for Evidence-Based Medicine (OCEBM) in an attempt to apply the classification to an animal study, it would likely be considered a form of preclinical evidence, which does not directly fit into the OCEBM levels of evidence that are more focused on clinical studies in humans. However, in cases of extreme necessity, it might be considered as level 5 evidence.

## Introduction

Noise is a significant harmful agent to hearing with occupational noise being the main cause of sensorineural hearing loss in adults. When noise is intense and the exposure to it is continuous, changes are developed in the internal structure of the cochlea, characterizing Noise Induced Hearing Loss (NIHL).^1^

Ordinance 19 of the Brazilian Ministry of Labor of 1998 describes NIHL as sensorineural auditory threshold shifts resulting from consistent occupational exposure to high sound pressure levels. It is irreversible, progressive and gradual according to the duration of exposure. It initially affects the auditory thresholds at one or more frequencies in the range of 3,000–6,000 Hz. The higher and lower frequencies, in general, take longer to be affected.^2^ NIHL leads to degeneration of the hair cells in the organ of Corti, and different mechanisms, such as mechanical stress, oxidative stress and the production of free radicals are pointed out as causing this alteration.^2^

The intensity and duration of exposure are crucial when conducting research on this subject, as low-intensity stimuli may fail to reach the levels necessary to induce a permanent threshold shift, while high-intensity stimuli risk causing acoustic trauma and compromise the observation of important auditory structures, as well as the analysis of audiological findings.

We found several studies in the literature involving noise exposures in animal models, some with the aim of studying beneficial effects of otoprotective agents, with short-term exposures^3^, ^4^, ^5^, ^6^, ^7^, ^8^, ^9^, ^10^, ^11^, ^12^, ^13^ that possibly generated temporary damage, which makes it difficult to analyze the results and may lead to wrong conclusions regarding the efficacy of otoprotective agents and measures.

Temporary Threshold Shift (TTS) is the result of high sound pressure levels exposure for any period of time capable of causing fatigue of auditory sensory cells,^14^, ^15^ leading to an increase in auditory thresholds that, after a period of rest, return to baseline thresholds.^16^ This acoustic phenomenon seems to occur through different mechanisms from those related to NIHL, where there is no total recovery of hearing thresholds, generating a Permanent Threshold Shift (PTS).^17^, ^18^, ^19^, ^20^, ^21^, ^22^ Research reports that thresholds shifts caused by noise exposures in experimental studies initially increase with the duration of exposure and eventually reach stability, known as plateau, characterizing the Asymptotic Threshold Shift (ATS).^18^, ^19^, ^20^, ^21^, ^22^

Considering that previous exposures may cause the phenomenon of “toughening”, which is characterized by the gradual reduction of ears susceptibility to suffer changes in post-exposure to noise thresholds, provided that they have received previous conditioning, it is necessary to distinguish sequential exposures with different noise doses from studies without previous exposure. Compound Threshold Shift (CTS) is defined as an auditory threshold shift resulting from sequential noise exposures at different doses and/or with rest periods between exposures.^23^

Acoustic trauma, on the other hand, is caused by short-term and high-intensity sounds, which can result in immediate, severe, and permanent hearing loss in which all structures of the ear can be injured. Although the auditory system has structures capable of “attenuating” the vibrations that reach the cochlea, sounds of strong intensity and short duration do not seem to allow this system to have “time to come into action”, occurring traumatic injury then.^24^

Studies on NIHL in animal models use Evoked Otoacoustic Emissions (EOAE) as a way to evaluate the functionality of outer hair cells, as this test allows to identify the onset of cochlear injuries, before any visible alteration in audiometry, and can detect cases of occupational hearing loss early.^25^, ^26^

The use of rats in hearing loss studies increased in the 1980s due to anatomical similarities with humans, and the reliability and repeatability in researching auditory thresholds, presenting a more viable model than guinea pigs and chinchillas. In studies on NIHL, Evoked Otoacoustic Emissions (EOAEs) are utilized to assess the functionality of outer hair cells, enabling early detection of cochlear lesions before audiometric changes are observed.^26^, ^27^

In this context, the aim of this study was to evaluate the effects of different noise doses on the Wistar rat animal model, using EOAEs to characterize the onset of TTS, the stabilization points of ATS, and the presence of PTS. This study aims to establish a guideline protocol based on noise doses, promoting uniformity and replicability of data in the literature.

## Methods

### Animals

Six male Wistar rats were acquired from the Vivarium of Uniceub College. The animals were housed in the Vivarium of the Faculty of Medicine of the University of Brasília and had free access to food and water. They were kept at a temperature of 22 °C with a 12 -h light-dark cycle. This study was approved by the Animal Use Ethics Committee ‒ SEI nº 23106.001211/2020-39 and executed in accordance with the guidelines of the National Institute of Health (NIH) and the National Council for the Control of Animal Experimentation (CONCEA). The animals underwent a health inspection process carried out by a veterinarian and had their weight recorded for calculation of anesthetic medication before hearing exams. Background noise levels where the animals were housed were below 60 Db SPL.

### Noise exposure

**Fig. 1:**
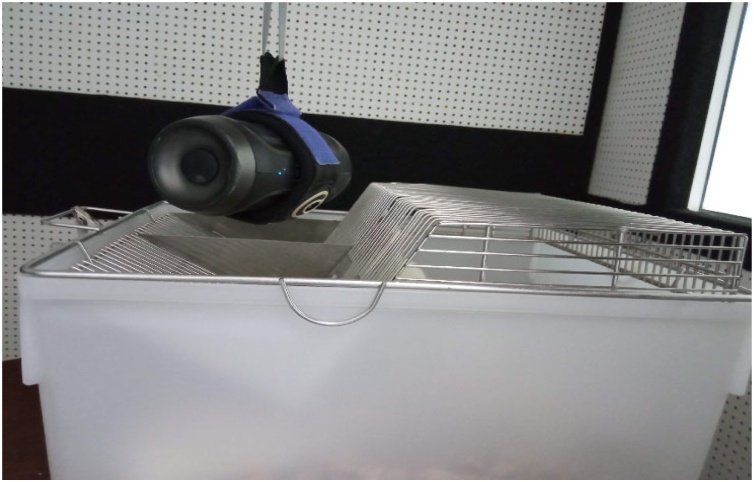
Position of the amplification source, used for noise exposure. Source: Developed by the author, 2023.

During the exposure, the animals were kept in a breeding box (Bonther Brand – 410 × 340 × 175 mm) and placed inside an acoustic booth (ReduSom Brand ‒ 1.00 × 1.00 × 1.80 m), lined with wood shavings but without environmental enrichment, such as tubes, food, and water, to avoid potential noise attenuation depending on the animals' positioning in the box. The sound source (JBL by Harman) was positioned 10 cm above the center of the box as illustrated in Fig. 1. Noise levels at the animals' ear height (3 cm above the bottom of the cage) were measured with a sound level meter (Instrutherm DEC ‒ 415) directly below the loudspeaker. Measurements near the edges of the cage at this height could show levels up to 3 dB SPL lower. The noise exposure was conducted in a separate room but within the animal housing facility, ensuring compliance with CONCEA regulations for animal handling during research. Noise was measured outside the booth, in the room designated for exposure, reaching a maximum intensity of 77 dB SPL. In the animal housing rooms, sound intensity was not affected during exposure, remaining below 60 dB SPL.

**Fig. 2:**
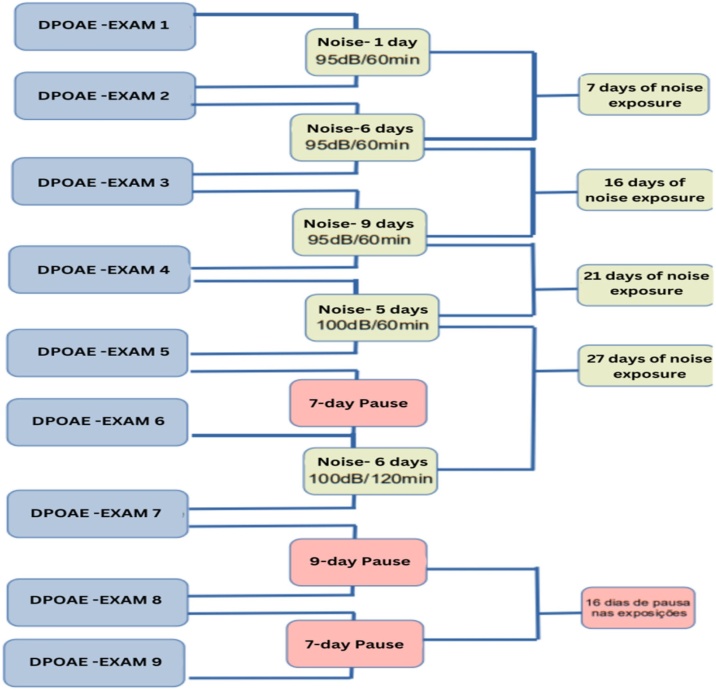
Flowchart of the protocol of noise exposures/pauses and exams performed. Source: Developed by the author, 2023.

For the exposure, a broadband white noise (Noise Generator – White noise) ranging from 1 to 10 kHz was used. The animals were subjected to sequential composite exposures at intensities of 95 dB SPL for 60 min, 100 dB SPL for 60 min, and 100 dB SPL for 120 min. The complete exposure protocol is illustrated in Fig. 2.

### Distortion product otoacoustic emissions

The first DPOAE exam was performed on all animals immediately after the general health inspection. For this, the animals were anesthetized with 5% isoflurane for induction and 3% for maintenance.^27^ The animals were covered so that their body temperature was maintained around 37 °C.

The exams were performed with an Evoked Otoacoustic Emissions device (EROSCAN® ‒ MAICO Diagnostics) and to obtain the distortion product (2F1‒F2), two pure tones were used in the ratio of F2/F1 = 1.22 presented at an average intensity of 65 dB SPL for f1 and 55 dB SPL for f2.^1^ Frequencies from 1.5 to 12 KHz were evaluated, and each test was performed twice. For the exams results, it was considered the average values of frequency amplitude and signal-to-noise ratio acquired in each of the two measurements performed. The normality of the variables was analyzed by the Kolmogorov-Smirnov test, while the homogeneity of the variances was analyzed by the Barttlet test. For comparisons between exposure doses, the *t*-test was used for parametric data and the Mann-Whitney test for non-parametric data. The data were expressed in tables containing the medians, maximum/minimum values. Significant differences were considered with *p* < 0.05. Analyses were performed using the Prism® 5 Software Package (GraphPad, USA, 2005).

## Results

8 ears were considered for the study, since there was no difference in laterality. Exam 1 was considered a reference and the analysis was based on the values found in the 9 DPOAE exams performed over 50 days of research, according to the research flowchart (Fig. 2).

In the analyses performed after 1, 7 and 15 days of noise exposure at an intensity of 95 dB for a period of 60 min (Exams 2, 3 and 4), no differences were observed between median frequencies amplitudes and S/N ratio in the frequencies searched, as shown in Table 1, Table 2.

**Table 1:** Median amplitude variation of DPOAEs in the control group comparison in examinations conducted on D0, D15, and D30.

	Control D0	Control D15		Control D15	Control D30		Control D0	Control D30	
AMP	Mean ± SD	Mean ± SD		Mean ± SD	Mean ± SD		Mean ± SD	Mean ± SD	
FREQ.	Median (Min‒Max)	Median (Min‒Max)	p	Median (Min‒Max)	Median (Min‒Max)	p	Median (Min‒Max)	Median (Min‒Max)	p
3	7.12 ± 6.40	9.00 ± 5.14	0.529	9.00 ± 5.14	10.19 ± 6.33	0.686	7.12 ± 6.40	10.19 ± 6.33	0.352
	5.50 (1.00–16.00)	9.50 (1.00–14.50)		9.50 (1.00–14.50)	12.25 (-1.50–17.50)		5.50 (1.00–16.00)	12.25 (-1.50–17.50)	
4	18.63 ± 5.59	20.81 ± 5.49	0.443	20.81 ± 5.49	20.56 ± 6.98	0.937	18.63 ± 5.59	20.56 ± 6.98	0.550
	19.75 (12.00–26.00)	22.15 (12.00–28.00)		22.15 (12.00–28.00)	22.75 (9.50–27.00)		19.75 (12.00–26.00)	22.75 (9.50–27.00)	
5	16.00 ± 3.66	19.04 ± 5.29	0.203	19.04 ± 5.29	17.60 ± 7.04	0.651	16.00 ± 3.66	17.60 ± 7.04	0.577
	16.00 (9.00–20.00)	19.90 (9.00–25.00)		19.90 (9.00–25.00)	20.15 (3.00–23.00)		16.00 (9.00–20.00)	20.15 (3.00–23.00)	
6	24.25 ± 3.88	24.38 ± 3.82	0.949	24.38 ± 3.82	24.38 ± 3.82	0.549	24.25 ± 3.88	24.38 ± 3.82	0.582
	24.75 (17.00–29.00)	24.75 (17.00–30.00)		24.75 (17.00–30.00)	25.00 (12.00–29.00)		24.75 (17.00–29.00)	25.00 (12.00–29.00)	
7	24.06 ± 3.35	24.94 ± 4.20	0.652	24.94 ± 4.20	23.13 ± 6.08	0.499	24.06 ± 3.35	23.13 ± 6.08	0.708
	24.50 (19.50–29.00)	24.75 (20.00–32.00)		24.75 (20.00–32.00)	23.75 (15.00–31.00)		24.50 (19.50–29.00)	23.75 (15.00–31.00)	
8	23.94 ± 2.51	24.13 ± 3.61	0.905	24.13 ± 3.61	23.63 ± 4.00	0.797	23.94 ± 2.51	23.63 ± 4.00	0.854
	24.00 (20.00–28.00)	23.75 (20.00–30.00)		23.75 (20.00–30.00)	24.25 (18.00–28.00)		24.00 (20.00–28.00)	24.25 (18.00–28.00)	
9	26.94 ± 2.00	26.88 ± 4.38	0.971	26.88 ± 4.38	26.81 ± 4.40	0.977	26.94 ± 2.00	26.81 ± 4.40	0.942
	27.00 (23.00–30.00)	27.00 (22.00–34.50)		27.00 (22.00–34.50)	28.00 (21.00–32.00)		27.00 (23.00–30.00)	28.00 (21.00–32.00)	
10	27.44 ± 4.53	26.13 ± 4.97	0.590	26.13 ± 4.97	25.81 ± 4.52	0.897	27.44 ± 4.53	25.81 ± 4.52	0.585
	28.25 (21.00–34.00)	26.00 (18.50–33.00)		26.00 (18.50–33.00)	27.00 (19.50–31.00)		28.25 (21.00–34.00)	27.00 (19.50–31.00)	
11	26.34 ± 4.98	22.63 ± 6.28	0.211	22.63 ± 6.28	24.50 ± 3.57	0.475	26.34 ± 4.98	24.50 ± 3.57	0.411
	26.50 (20.00–34.00)	22.00 (13.50–32.00)		22.00 (13.50–32.00)	26.00 (19.00–29.50)		26.50 (20.00–34.00)	26.00 (19.00–29.50)	
12	23.88 ± 7.51	22.98 ± 5.27	0.785	22.98 ± 5.27	22.38 ± 3.14	0.786	23.88 ± 7.51	22.38 ± 3.14	0.610
	22.50 (16.00–36.50)	24.00 (16.00–30.50)		24.00 (16.00–30.50)	22.75 (17.00–26.00)		22.50 (16.00–36.50)	22.75 (17.00–26.00)	

SD, Standard Deviation; DPOAE, Distortion Product Otoacoustic Emissions.

Statistical significance determined by the T-test/Mann-Whitney test, for p < 0.05.

**Table 2:** Median variation of the Signal-to-Noise Ratio (SNR) of DPOAEs in the control group comparison in examinations conducted on D0, D15, and D30.

	Control D0	Control D15		Control D15	Control D30		Control D0	Control D30	
SNR	Mean ± SD	Mean ± SD		Mean ± SD	Mean ± SD		Mean ± SD	Mean ± SD	
FREQ.	Median (Min‒Max)	Median (Min‒Max)	p	Median (Min‒Max)	Median (Min‒Max)	p	Median (Min‒Max)	Median (Min‒Max)	p
3	23.06 ± 6.52	23.69 ± 5.35	0.837	23.69 ± 5.35	27.44 ± 7.37	0.263	23.06 ± 6.52	27.44 ± 7.37	0.229
	20.75 (15.5–35.00)	24.25 (16.50–30.50)		24.25 (16.50–30.50)	29.25 (17.50–35.00)		20.75 (15.5–35.00)	29.25 (17.50–35.00)	
4	35.25 ± 6.39	37.00 ± 6.43	0.593	37.00 ± 6.43	35.18 ± 8.10	0.625	35.25 ± 6.39	35.18 ± 8.10	0.983
	35.00 (26.5–44.00)	37.50 (27.00–45.00)		37.50 (27.00–45.00)	37.85 (22.50–44.70)		35.00 (26.5–44.00)	37.85 (22.50–44.70)	
5	33.44 ± 5.39	34.48 ± 5.22	0.701	34.48 ± 5.22	35.35 ± 6.99	0.781	33.44 ± 5.39	35.35 ± 6.99	0.550
	35.25 (24.50–40.00)	32.50 (27.30–44.00)		32.50 (27.30–44.00)	37.25 (23.00–43.50)		35.25 (24.50–40.00)	37.25 (23.00–43.50)	
6	43.04 ± 2.10	42.75 ± 3.38	0.773	42.75 ± 3.38	42.53 ± 6.25	0.929	43.04 ± 2.10	42.53 ± 6.25	0.923
	43.50 (40.50–46.00)	42.50 (37.00–49.00)		42.50 (37.00–49.00)	45.00 (32.00–49.00)		43.50 (40.50–46.00)	45.00	
7	43.74 ± 2.50	43.88 ± 4.04	0.707	43.88 ± 4.04	43.13 ± 6.08	0.775	43.74 ± 2.50	43.13 ± 6.08	0.971
	44.00 (40.00–46.50)	43.85 (37.30–49.50)		43.85 (37.30–49.50)	43.75 (35.00–51.00)		44.00 (40.00–46.50)	43.75 (35.00–51.00)	
8	43.94 ± 2.51	44.06 ± 3.53	0.936	44.06 ± 3.53	43.50 ± 3.76	0.762	43.94 ± 2.51	43.50 ± 3.76	0.788
	44.00 (40.00–48.00)	43.75 (40.00–50.00)		43.75 (40.00–50.00)	44.25 (38.00–47.50)		44.00 (40.00–48.00)	44.25 (38.00–47.50)	
9	46.91 ± 1.99	46.94 ± 4.30	0.988	46.94 ± 4.30	46.41 ± 4.25	0.809	46.91 ± 1.99	46.41 ± 4.25	0.768
	47.00 (43.00–50.00)	47.00 (42.50–54.50)		47.00 (42.50–54.50)	47.65 (40.00–52.00)		47.00 (43.00–50.00)	47.65 (40.00–52.00)	
10	47.28 ± 4.48	45.88 ± 5.13	0.570	45.88 ± 5.13	44.94 ± 5.77	0.736	47.28 ± 4.48	44.94 ± 5.77	0.381
	47.10 (41.00–54.00)	46.00 (37.50–53.00)		46.00 (37.50–53.00)	47.00 (34.00–50.00)		47.10 (41.00–54.00)	47.00 (34.00–50.00)	
11	45.65 ± 5.34	43.19 ± 6.29	0.413	43.19 ± 6.29	44.53 ± 3.45	0.606	45.65 ± 5.34	44.53 ± 3.45	0.625
	45.25 (40.00–54.00)	43.50 (31.50–52.00)		43.50 (31.50–52.00)	45.75 (38.50–49.50)		45.25 (40.00–54.00)	45.75 (38.50–49.50)	
12	43.60 ± 7.60	40.63 ± 8.17	0.465	40.63 ± 8.17	40.81 ± 3.74	0.953	43.60 ± 7.60	40.81 ± 3.74	0.368
	41.40 (36.00–56.50)	41.00 (25.50–50.50)		41.00 (25.50–50.50)	41.50 (36.00–46.00)		41.40 (36.00–56.50)	41.50 (36.00–46.00)	

SD, Standard Deviation; DPOAE, Distortion Product Otoacoustic Emissions.

Statistical significance determined by the T-test/Mann-Whitney test, for p < 0.05.

After increasing the intensity of noise exposure to 100 dB in sequential exposure, for 60 min, for 5 days (Exam 5), it was possible to verify a difference between median frequencies amplitudes at the frequencies of 8 KHz (E1 = 29.25 and E5 = 26.25 and *p* =  0.014); 9 KHz (E1 = 30.00 and E5 = 27.00 and *p* =  0.039); 10 KHz (E1 = 29.75 and E5 = 25.25 and *p* = 0.024); 11 KHz (E1 = 26.75 and E5 = 20.00 and *p* =  0.004) and 12 KHz (E1 = 36.00 and E5 = 24.00 and *p* <  0.000) and between median signal-to-noise ratio of examinations 1 and 5 at frequencies of 8 KHz (E1 = 49.25 and E5 = 46.00 and *p* =  0.020); 10 KHz (E1 = 48.50 and E5 = 45.25 and *p* =  0.048); 11KHz (E1 = 46.75 and E5 = 40.00 and *p* = 0.006) and 12 KHz (E1 = 56.00 and E5 = 44.00 and *p* <  0.001). After a 7-day break in noise exposure, there was a recovery in auditory thresholds (Exam 6), as shown in Table 1, Table 2.

After increasing the exposure time to 120 min at an intensity of 100 dB for 6 days, it was possible to observe a difference between the median frequency amplitude, at frequencies of 3 KHz (E1 = 9.25 and E7 = 6.75 and *p* = 0.034); 8 KHz (E1 = 29.25 and E7 = 23.75 and *p* =  0.000); 9 KHz (E1 = 30.00 and E7 = 22.50 and *p* = 0.000); 10 KHz (E1 = 29.75 and E7 = 22.00 and *p* = 0.000); 11 KHz (E1 = 26.75 and E7 = 19.00 and *p* =  0.003) and 12 KHz (E1 = 36.00 and E7 = 19.75 and *p* <  0.000) and between the median signal-to-noise ratio, at frequencies of 8 KHz (E1 = 49.25 and E7 = 42.50 and *p* =  0.001); 9 KHz (E1 = 48.25 and E7 = 42.50 and *p* =  0.001); 10 KHz (E1 = 48.50 and E7 = 41.75 and *p* < 0.000); 11 KHz (E1 = 46.75 and E7 = 39.00 and *p* = 0.003) and 12 KHz (E1 = 56.00 and E7 = 39.50 and *p* <  0.001. This alteration remained evident after 9 days of exposure pause, and a difference was observed between the median frequency amplitude of exams 1 and 8 at frequencies of 4 KHz (E1 = 21.25 and E8 = 18.50 and *p* = 0.011); 5 KHz (E1 = 20.50 and E8 = 18.50 and *p* =  0.002); 8 KHz (E1 = 29.25 and E8=26.00 and *p* =  0.006); 9 KHz (E1 = 30.00 and E8 = 24.50 and *p* = 0.004); 10 KHz (E1 = 29.75 and E8 = 22.00 and *p* =  0.000); 11 KHz (E1 = 26.75 and E8 = 22.50 and *p* =  0.013) and 12 KHz (E1 = 36.00 and E8 = 24.50 and *p* = 0.000) and between the medians signal-to-noise ratio at the frequencies of 5 KHz (E1 = 38.50 and E8 = 34.50 and *p* =  0.430); 8 KHz (E1 = 49.25 and E8 = 46.00 and *p* = 0.010); 9 KHz (E1 = 48250 and E8 = 42.50 and *p* = 0.011); 10 KHz (E1 = 48.50 and E8 = 42.00 and *p* <  0.001); 11 KHz (E1 = 46.75 and E8 = 40.50 and *p* = 0.007) and 12 KHz (E1 = 56.00 and E8 = 42.50 and *p* = 0.000). Then, after 16 day of exposure pause, a difference was observed between the medians frequency amplitude of examinations 1 and 9 at frequencies of KHz (E1 = 29.25 and E9 = 26.25 and *p* = 0.002); 9 KHz (E1 = 30.00 and E9 = 22.25 and *p* = 0.016) and 12 KHz (E1 = 36.00 and E9 = 27.50 and *p* = 0.004, as well as between the medians the signal-to-noise ratio of examinations 1 and 9 at frequencies of 8 KHz (E1=49.25 and E9426.25 and *p* =  0.005); 9 KHz (E1 = 48.25 and E9 = 43.25 and *p* =  0.019); 11 KHz (E1 = 46.75 and E9 = 43.25 and *p* = 0.047) and 12 KHz (E1 = 56.00 and E9 = 46.75 and *p* = 0.004). This evolution can be seen in Table 1, Table 2.

**Fig. 3:**
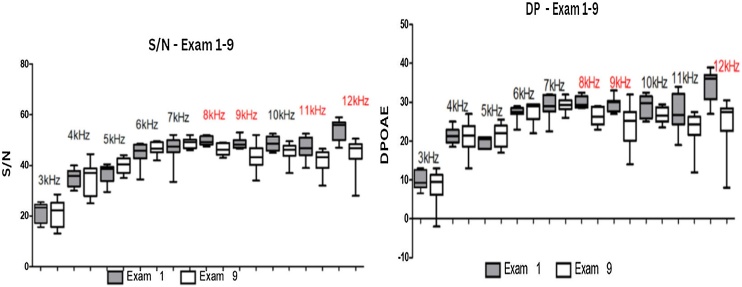
Boxplot illustrating the variation of the signal amplitude (A) and the signal-to-noise ratio (B) at frequencies from 3 to 12 KHz obtained between the first and ninth exam. Frequencies with statistically significant results are highlighted in red. Source: developed by the author, 2023.

Fig. 3 presents the results of DPOAE signal amplitude and S/N ratio values after a 16-day pause in exposure. A noticeable reduction in median values is observed for both signal amplitude and S/N ratio compared to the reference test.

**Fig. 4:**
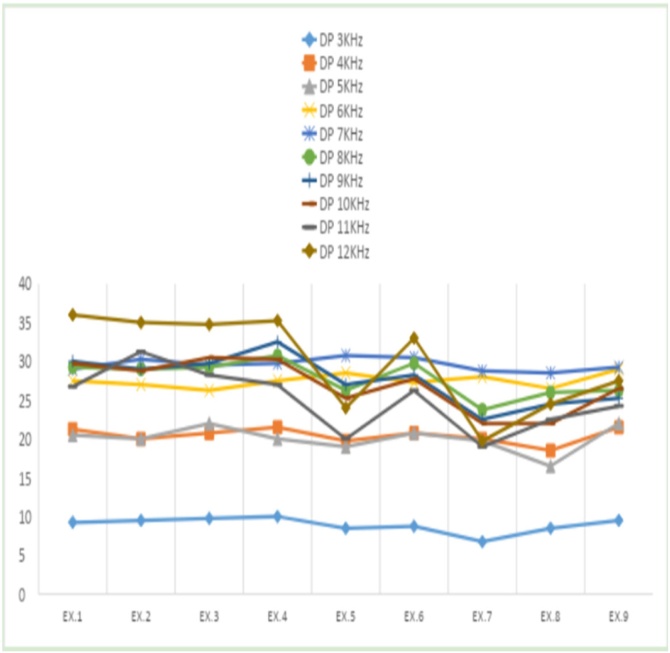
Median frequency amplitude from 3 to 12 KHz, from exam 1 to exam 9. Source: Developed by the author, 2024.

**Fig. 5:**
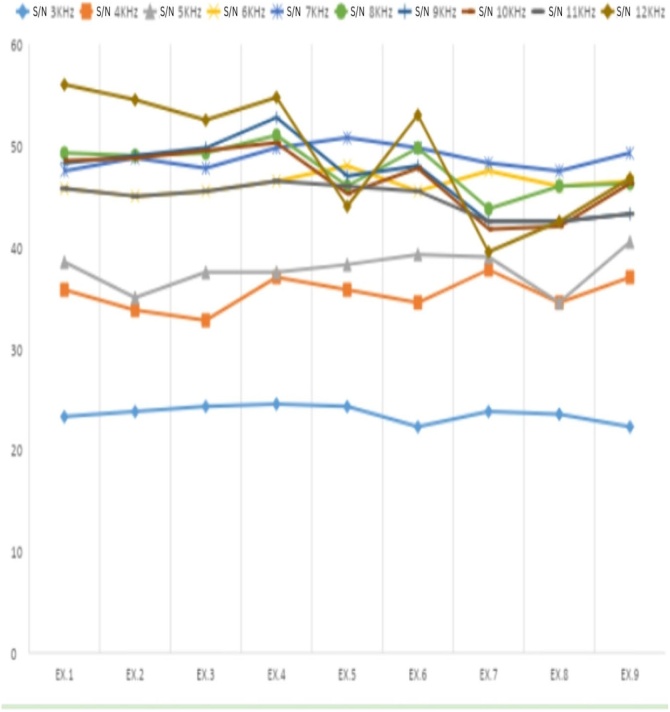
Median S/N ratio at frequencies 3 to 12 KHz, from exam 1 to exam 9. Source: Developed by the author, 2024.

Fig. 4, Fig. 5 display the shifts in median values for signal amplitude and signal-to-noise ratio over the 50-day study period. The variable X represents signal amplitude, while Y corresponds to the S/N ratio.

## Discussion

This study explored the effects of noise exposure on Wistar rats, chosen for their anatomical similarity to humans. The hearing range of rats varies from approximately 250 Hz to 80 kHz, with the greatest sensitivity between 8 and 38 kHz. The rat cochlea, with about two and a half turns, presents an arrangement of inner and outer hair cells similar to that found in humans, which reinforces its suitability as a model for auditory studies.^28^, ^29^, ^30^ We observed that exposures to 100 Db SPL for 60 min over five days resulted in temporary auditory threshold shifts, returning to baseline levels after seven days of rest. In contrast, exposures to 100 dB SPL for 120 min over six days induced permanent changes at specific frequencies.

The main consequence of Noise-Induced Hearing Loss (NIHL) is damage to the outer hair cells of the cochlea, although the exact mechanism remains unclear. Evidence suggests that the generation of Reactive Oxygen Species (ROS) during and after noise overexposure can activate stress signaling pathways, leading to cellular damage, apoptosis, or necrosis.^31^, ^32^ Additionally, noise can affect the synapses of hair cells and neurons, even when these remain viable and recover normal function. Research indicates that the loss of synapses and afferent terminals due to exposure is rapid and permanent, while the loss of spiral ganglion neurons is slower.^33^, ^34^ Thus, synapse and neural loss can amplify the functional consequences of noise exposure, reducing the auditory nerve's ability to encode signals faithfully, even without an evident threshold sensitivity loss.^35^, ^36^

Several animals studies^18^, ^23^, ^25^, ^26^, ^27^, ^37^, ^38^, ^39^, ^40^, ^41^ have been conducted to analyze the effects of otoprotective substances concomitant with noise exposure, however, it was not possible to establish a standardized protocol for the characteristics of these exposures. There is a wide range of applied protocols, with noise intensities ranging from 80 dB to 126 dB, daily exposure time ranging from 25 min to 8 h, and number of days ranging from 1 to 108 days.^3^, ^4^, ^8^, ^10^^,^^13^, ^23^, ^42^, ^43^ The intensity of 100 dB has been most commonly used to cause NIHL^13^, ^32^, ^38^, ^42^^,^^44^, ^45^, ^46^ and we found two studies reporting acoustic trauma with exposures at 105 dB for 2 and 4 h.^9^, ^12^

Our study focused on sequential exposures to different noise intensities. Initially, the animals were exposed to 95 dB SPL for 6 min, followed by exposures of 100 dB SPL for 60 min, and finally, 100 dB SPL for 120 min. These conditions were chosen to explore the phenomenon of “toughening”, where prior exposures can condition the auditory system, reducing susceptibility to subsequent damage. It was observed that after the first exposure of 95 dB SPL, there was no significant change in auditory thresholds, a result consistent with that reported by Cappaert, who also found no alterations after exposures to 90 and 100 dB SPL for up to five consecutive days.^42^ However, a study conducted by Chen reported discrete changes in thresholds after exposures to lower intensities,^23^ indicating that exposure time may play a crucial role in inducing auditory changes.

In this study, after exposure to 100 dB SPL for 60 min over 5 days, it was possible to verify changes in auditory thresholds. Significant alterations were found in both the frequency amplitude and the signal-to-noise ratio of DPOAEs, in the frequencies of 8–12 kHz, corroborating with the results found in other studies.^15^, ^23^, ^38^, ^40^^,^^42^, ^44^ It is noteworthy that this threshold shift was temporary, as after 7 days of rest, there was a return to baseline thresholds. Our findings align with those found by Mannström with different noise intensities, between 101 and 104 dB, which were considered temporary after tests conducted with two weeks of acoustic rest.^15^ However, by extending the exposure to 100 dB SPL for 120 min over 6 days, we found permanent changes at frequencies of 8, 9, and 12 KHz, after a 16-day pause in exposures, consistent with the study of Melnick, which suggests that permanent threshold shifts are only verifiable after at least 15 days of rest from exposures, without a return to baseline thresholds.^19^ It is important to mention that our study made significant progress by employing sequential exposures at different noise rates, which allowed these changes to be characterized as Compound Threshold Shift (CTS).^23^

The identification of a “plateau” in threshold shift after 6 days of noise exposure suggests that prolonged exposure to elevated levels may not result in additional changes in auditory thresholds. Researchers report that noise-induced hearing loss initially increases with the duration of exposure above a critical level. However, once the exposure duration exceeds 18 and/or 24 h, the hearing loss reaches a plateau defined as Asymptotic Threshold Shift (ATS).^18^, ^20^, ^21^, ^22^ This phenomenon was found and studied by Clark after 5 consecutive days of noise exposure, which aligns with our research.^18^

When defining protocols for experimental studies involving high levels of sound pressure exposure, it is essential that researchers understand the specific characteristics of the noise employed in their studies, while aligning this choice with the methods used for diagnosing hearing loss. Venet highlighted this fact by describing that the appearance of auditory changes in otoacoustic emissions and ABR tests usually occur one octave higher than the noise used. In our study, we used white noise with a wide spectrum of frequencies, and auditory changes were found between 8 and 12 KHz.

This study was conducted in accordance with animal welfare guidelines. The choice of a single experimental group may limit the extrapolation of the results, but it was a strategy adopted to balance ethical requirements and research objectives. It is important to consider that while our findings offer valuable insights into the dynamics of Noise-Induced Hearing Loss (NIHL), direct application to humans requires caution.

Studies analyzing NIHL in animals are generally conducted in the short term with very high sound levels. A study conducted on temporary and permanent changes in auditory thresholds in animals criticized this form of analysis. According to the authors, this format hinders the translation of information to humans, for whom NIHL is typically the result of several years of exposure. Short exposure is convenient for inducing ear damage in basic studies of morphology and function, but it is of lesser value for understanding noise-induced hearing loss in humans.^18^

There are also studies that, to analyze the effects of noise overexposure, involve very intense exposures that permanently damage the cochlea and can lead to altered transmission of acoustic information along the central auditory pathway.^27^ Traumatic noise exposure typically results in acute and chronic changes in the auditory system and hinders the correlation of threshold change with NIHL.

NIHL poses a significant risk to both occupational and public health in general. Considering the occurrence of NIHL based on studies with short exposures or even those that do not subject animals to a rest period before proving the occurrence of prolonged and permanent NIHL can be a dangerous path when science seeks to translate results from experimental animal studies to humans.

Currently, ongoing research in our laboratory will conduct complementary examinations such as Brainstem Auditory Evoked Response (BERA), histological analysis, and transmission electron microscopy, which may provide more comprehensive data and elucidate the effects of Noise-Induced Hearing Loss (NIHL) throughout the auditory system. It is essential for experimental protocols to continue evolving, aiming not only at the precise characterization of auditory changes but also at identifying potential interventions that can prevent or mitigate the effects of noise.

## Conclusion

This study aimed to assess the effects of different noise doses in Wistar rats and demonstrated, through DPOAEs, that 15 days of exposure to white noise for 60 min at an intensity of 95 dB did not result in a threshold shift, even temporarily. A temporary threshold shift was observed after 5 days of exposure to 100 dB for 60 min. However, stability and a permanent threshold shift were only achieved when the daily exposure time was increased to 120 min, with a “plateau” in the threshold shift after 6 days of exposure. Given the sequential exposure to varying noise doses, these changes are characterized as Compound Threshold Shifts (CTS).

## Funding

This research received financial aid from a scholarship according to Edict DPG nº 0011/2022.

## Declaration of competing interest

The authors declare no conflicts of interest.
